# Cystic Fibrosis Related Liver Disease—Another Black Box in Hepatology

**DOI:** 10.3390/ijms150813529

**Published:** 2014-08-04

**Authors:** Katharina Staufer, Emina Halilbasic, Michael Trauner, Lili Kazemi-Shirazi

**Affiliations:** 1 Division of Transplantation, Department of Surgery, Medical University of Vienna, Währinger Gürtel 18-20, A-1090 Vienna, Austria; 2 Division of Gastroenterology and Hepatology, Department of Internal Medicine III, Medical University of Vienna, Währinger Gürtel 18-20, A-1090 Vienna, Austria; E-Mails: Emina.Halilbasic@meduniwien.ac.at (E.H.); Michael.Trauner@meduniwien.ac.at (M.T.); Lili.Kazemi-Shirazi@meduniwien.ac.at (L.K.-S.)

**Keywords:** cystic fibrosis, bile acid transporters, gut liver axis, non-invasive diagnostics, cholestasis

## Abstract

Due to improved medical care, life expectancy in patients with cystic fibrosis (CF) has veritably improved over the last decades. Importantly, cystic fibrosis related liver disease (CFLD) has become one of the leading causes of morbidity and mortality in CF patients. However, CFLD might be largely underdiagnosed and diagnostic criteria need to be refined. The underlying pathomechanisms are largely unknown, and treatment strategies with proven efficacy are lacking. This review focuses on current invasive and non-invasive diagnostic standards, the current knowledge on the pathophysiology of CFLD, treatment strategies, and possible future developments.

## 1. Introduction

Cystic fibrosis (CF) is the most frequent fatal autosomal recessive disorder in Caucasians. It results from mutations within the cystic fibrosis transmembrane conductance regulator (CFTR) comprising more than 1900 known mutations [1]. The most common *CFTR* mutation, *F508del*, can be found in about 68% of patients [2]. *CFTR* encodes for a protein that is found in epithelial cells of lungs, sweat glands, pancreas, intestine and liver. It belongs to the family of ATP-binding cassette (ABC) transporters and functions as a cAMP-dependent chloride channel facilitating a Cl^−^/HCO_3_^−^ exchange on exocrine epithelia [3]. Thereby, it maintains an alkaline pH and dilutes fluid secretions [4]. Defects of CFTR lead to dehydration of secretions and hyperviscous mucus causing a multisystem disease with major affection of the respiratory, gastrointestinal, and hepatobiliary tract.

Advances in patient care including transplantation have led to a strong increase of life expectancy in patients with CF with an estimated median survival of >50 years in patients born in the UK in 2000 and thereafter [5]. Albeit lung disease remains the main cause of morbidity and mortality, the changing demographics of CF necessitate an increased focus also on other organ manifestations.

CF-related liver disease (CFLD) was recently shown to be an independent risk factor for mortality and lung transplantation, and has become one of the leading causes of death among patients with CF [6].

## 2. Clinical Presentation of CFLD

The absence of functioning CFTR may lead to cholestatic liver disease with reduced bile flow and focal biliary obstruction causing periductal inflammation, proliferation and finally periportal fibrosis. The morphological findings are diverse and comprise liver alterations ranging from steatosis to primary sclerosing cholangitis (PSC)–like bile duct lesions, cholestasis, biliary cirrhosis, and alterations of the gall bladder (*i.e*., microgallbladder). About 40% of CF patients show abnormal imaging results (ultrasound, magnetic resonance imaging, or computed tomography). Up to 60% of patients present with signs of steatosis, and up to 30% develop advanced CFLD including (focal) biliary cirrhosis and portal hypertension [7,8].

## 3. Diagnosis of CFLD

Diagnosis of CFLD is indeed challenging due to the often asymptomatic presentation and wide spectrum of manifestations. For the diagnosis of CFLD, currently, an annual review including assessment of liver function tests as well as a physical examination are recommended to detect elevated liver enzymes and hepatomegaly and/or splenomegaly [8]. Only in case of pathological findings (hepatomegaly with or without splenomegaly and/or elevated liver enzymes) ultrasound is advocated by some authors [8]. However, our practice is to perform annual ultrasound of the abdomen in all patients with CF, as ultrasound is non-invasive, cost-effective and highly valuable for the diagnosis of hepatic steatosis, cirrhosis and complications of portal hypertension such as ascites and splenomegaly. Yet, ultrasound cannot reliably exclude early liver disease [9] and disparity between results of ultrasound and liver enzymes was observed in up to 24% of CF patients [10,11]. Several studies have demonstrated that clinical evaluation, serum liver enzymes and ultrasound are imprecise for detecting the presence and predicting the progression of liver disease [10,11,12,13]. Thus, for patients in whom diagnosis is still inconclusive after standard work-up (see above) including exclusion of other causes of liver disease (viral, autoimmune, metabolic, genetic) percutaneous liver biopsy representing the diagnostic gold standard, can be performed [8]. Although liver biopsy is invasive, complication rates after liver biopsy in CF patients seem to be low even when dual pass liver biopsies are performed (no bleeding, hospital admission, prolonged pain or surgery [9,13]. However, a substantial sampling error due to typically focally distributed lesions in CFLD can complicate diagnostic algorithms. Since all established diagnostic measures cannot reliably preclude CF related liver involvement, there is an urgent clinical need for advanced diagnostic tools. Minimally- and non-invasive methods for assessment of liver fibrosis have recently become a focus of interest in the diagnosis and management of CFLD.

### 3.1. Serum Parameters and Emerging Biomarkers

Serum markers offer an attractive and cost-effective approach. The impact of abnormalities of serum liver enzymes, however, is limited in screening for CFLD. Elevation of transaminases (aspartate amino transferase (AST), alanine amino transferase (ALT)) or cholestasis parameters (alkaline phosphatase (AP), gamma glutamyl transferase (GGT)) are frequently mild or intermittently present, sensitivity and specificity are low and abnormalities usually do not correlate with histological findings. In a study of 43 patients with CF, all of whom underwent liver biopsy, an elevated GGT or ALT had a low sensitivity of 50% and 52% and a specificity of 74% and 77% for detection of liver fibrosis, respectively [12]. Liver enzymes may be even normal although multilobular biliary cirrhosis is present [8]. In a recent prospective study of 40 children with CF with dual-pass liver biopsy in all patients and up to 12 years follow-up, 25% of those with high grade fibrosis (F3–4) had normal ALT levels [13] confirming findings of previous studies.

However, serum markers of liver fibrosis may be useful for early detection of patients with CFLD and identify patients at risk for progression of liver disease. In a case control study, Pereira *et al.* demonstrated that elevated tissue inhibitor of matrix metalloproteinase (TIMP)-1, collagen (CL)-IV, and prolylhydroxylase (PH) may be indicators of hepatic fibrogenesis in CF, and increased TIMP-1, and PH may be early markers of CFLD [14]. Nevertheless, validation studies are lacking.

Rath *et al.* investigated the regulation of 220 different serum proteins for the diagnosis of CFLD [15]. TIMP-4 and endoglin were increased in patients with CFLD and exhibited a high diagnostic accuracy (sensitivity 65% and 71% and specificity 82% and 71%, respectively). Their sensitivity was further increased by combinational use of transient elastography (TE) (sensitivity of TE and endoglin: 88%, TE and TIMP-4: 100%) [15].

### 3.2. Non-Invasive Imaging Techniques for Diagnosing CFLD

Transient elastography (FibroScan^®^, Echosense, Paris, France), Acoustic Radiation Force Impulse (ARFI, Siemens AG, Erlangen, Germany) and magnetic resonance imaging (MRI) are advancing along the route to clinical routine.

FibroScan^®^, measuring the stiffness of hepatic parenchyma using both ultrasound and low-frequency elastic waves produced by a specialized ultrasound vibrator applied to the body wall [16], is considered a reliable method for the diagnosis of significant fibrosis and cirrhosis. In a study on 66 CF patients liver stiffness as assessed by FibroScan^®^ was significantly increased in patients with clinical, biochemical and ultrasound evidence for CFLD [17]. In a prospective study of 134 consecutive patients with CF and 31 controls, all pancreatic sufficient patients and controls had normal transient elastography TE values (<7 kPa) and significantly higher elasticity values were noted in males [18], consistent with findings by several investigators that male gender and pancreatic insufficiency are independent risk factors for severe CFLD.

ARFI imaging combines conventional ultrasonography with evaluation of local liver stiffness. Regions of evaluation are chosen by ultrasound, allowing avoidance of anatomic obstacles [19,20]. Several studies have assessed the use of ARFI in the evaluation of CFLD [21,22,23,24,25,26]. Shear Wave Velocity increased progressively from patients with no evidence of CFLD to patients with CFLD with no evidence of portal hypertension to those with signs of portal hypertension [21].

Right liver lobe ARFI, TE, and laboratory based fibrosis indices correlated with each other and reliably detected CFLD related liver cirrhosis in a prospective study of 55 adult CF patients evaluating TE and ARFI simultaneously. However, TE had a higher rate of invalid measurements than ARFI in patients without CFLD. Both TE and ARFI could not significantly discriminate between non-cirrhotic CFLD and CF without liver disease [22]. Thus, TE and ARFI seem to be useful in distinguishing between CF patients with liver cirrhosis and earlier stages of CFLD. Elastography values in CFLD cirrhosis differed from cut-off values in patients with alcoholic cirrhosis or those reported in viral hepatitis induced cirrhosis, strengthening the assumption, that elastography values depend on the etiology of liver disease [22].

MRI technology can be used both for measurement of liver stiffness (by Magnetic Resonance Elastography; MRE; no data available in CF), and evaluation of liver parenchyma and bile duct alterations [27], but is associated with substantially higher costs and examination times than TE or ARFI.

Thus far, data on MRI-based imaging studies in CFLD are scarce. In a study from 1999 in 27 adult CF patients (1.5 Tesla, no contrast enhanced imaging) magnetic resonance cholangiography (MRC) in comparison to ultrasound was able to detect cholangiopathy in a higher proportion of patients [28]. ^1^H Magnetic resonance spectroscopy (MRS), a technique to differentiate simple steatosis from potentially progressive steatohepatitis, characterizing the intrahepatic lipid pattern has not been performed in CF thus far.

To obtain a more comprehensive morphological picture of the disease MRS, MRC using a liver-specific contrast agent to assess the hepatobiliary excretory function, TE/ARFI, as well as ultrasound might improve non-invasive diagnostics to identify patients at risk for CFLD and disease progression.

## 4. Differential Diagnosis of CFLD

### 4.1. Non-Alcoholic Fatty Liver Disease

Non-alcoholic fatty liver disease (NAFLD) is now considered one of the most important causes of liver disease in Western countries, ranging from simple steatosis to steatohepatitis (NASH) and cirrhosis. As the hepatic manifestation of the metabolic syndrome, NAFLD is typically encountered in individuals with insulin resistance. Although steatosis in CF does not seem to be related to a CFTR secretory defect, but has been associated with selective nutritional deficiencies and altered phospholipid metabolism, its relevance as a risk factor for the development of steatohepatitis and progression to cirrhosis in CF patients is unclear.

However, a high proportion of CF patients develops CF related diabetes (CFRD) [29], which shares features of type I (insulin deficiency) and type II (insulin resistance) diabetes. Thus the high prevalence of 20%–60% of steatosis described in patients with CF [30] might indeed represent NAFLD [31]. The pathogenic and histomorphologic relation of CFLD to NAFLD has not been investigated so far.

### 4.2. Primary Sclerosing Cholangitis

Primary sclerosing cholangitis (PSC) is a chronic progressive cholestatic disorder of unknown etiology characterized by inflammatory bile duct alterations resembling those seen in CF [32]. Mutations in *CFTR*, which in general is highly expressed on biliary epithelial cells [33], have been detected in PSC patients [34,35], although other studies have revealed discordant results [36]. In addition, 60% to 80% of PSC patients suffer from inflammatory bowel disease (IBD) [37], and an association with IBD has also been reported in CF [38]. Both CF and PSC with IBD are associated with an extremely high rate of colorectal cancer [37,39] adding to the similarities between these two disease entities. Bile acids with their chemical properties and capacities to activate receptors, such as Farnesoid X receptor (FXR) and G-protein coupled bile acid receptor 1 (TGR5), could be the common denominator for these analogies between PSC and CFLD.

However, in contrast to CF, which only affects the intrahepatic bile ducts, PSC can involve the intrahepatic, extrahepatic bile ducts or both and leads to concentric obliterative fibrosis with bile duct strictures leading to disruption of biliary secretion and finally biliary cirrhosis [37,40].

### 4.3. Secondary Sclerosing Cholangitis

Secondary sclerosing cholangitis (SSC) comprises a group of cholangiopathies which are morphologically similar to PSC but originate from a known pathological process, suggesting that widely different insults may give rise to a similar pattern of biliary disease [41]. Indeed. CFLD itself may be a form of SSC. Thus, the diagnosis of PSC requires the exclusion of secondary causes of sclerosing cholangitis and careful history-taking is key. Differentiating between PSC and SSC or the various causes of SSC and SSC can be particularly difficult, since common causes of SSC such as previous bile duct surgery, cholangiolithiasis, recurrent pancreatitis, recurrent infectious cholangitis, portal biliopathy (bile duct and gallbladder wall anomalies seen in patients with portal hypertension), or ischemia-induced bile duct lesions, also called ischemic cholangitis [42] (e.g., after lung transplantation with prolonged ECMO-therapy in CF) may be present in the same patient.

Noteworthy, IgG4-associated cholangitis (IAC), another cause of SSC, is a steroid-responsive cholangiopathy, which is biochemically and cholangiographically indistinguishable from PSC, but is often associated with autoimmune pancreatitis and other fibrosing conditions (e.g., retroperitoneal fibrosis). It is characterized by elevated IgG4-levels and infiltration of IgG4 positive plasma cells in bile ducts and liver tissue. Unlike PSC, it mainly affects elder individuals and has a good long-term prognosis [43,44]. The prevalence of elevated IgG4 levels in CFLD has not been investigated thus far.

## 5. The Pathomechanism of CFLD

The distinct pathomechanisms underlying CFLD, yet, have remained elusive. It is very likely, that a combination of *CFTR* genotype, individual susceptibility to environmental factors and the bi-directional influence of affected organ systems in combination lead to the development and progression of CFLD, rather than one single hit [45,46]. Several risk factors for occurrence or progression of CFLD have been defined, such as younger age at CF diagnosis, male sex, history of meconium ileus, exocrine pancreatic insufficiency, CF related diabetes (CFRD), low body mass index (BMI), growth failure, abnormal nutritional parameters, chronic lung colonization with Burkholderia cepacia complex, cumulative amount of antibiotics, but also TGFβ polymorphisms [47,48].

### 5.1. Genetic Factors in CFLD

*CFTR* mutations are divided into class I, II, III, IV, V, and VI depending on the quality of CFTR aberration [49,50]. *CFTR* mutations might affect biosynthesis (class I), protein maturation (class II, including *F508del* mutation), Cl^−^ regulation and gating (class III), Cl^−^ conductance (class IV), protein stability (class V), and turnover of the CFTR channel at the cell surface (class VI) [49,50]. Of note, the *CFTR* phenotype is highly heterogeneous among individual patients, and *CFTR* genotype-phenotype correlations are generally weak [48]. So-called severe mutations (classes I, II, or III) presenting with higher morbidity and mortality correlate well with pancreatic insufficiency (>95%), meconium ileus (>20%), higher sweat chloride levels (≥60 mmol/L) [51], younger age (<1 year) at diagnosis, and liver disease (in 3%–5%) [52]. It is to mention, that history of meconium ileus, as well as exocrine pancreatic insufficiency and younger age at diagnosis, all associated with more severe *CFTR* mutation classes, have been identified as risk factors for the development of CFLD [47,48].

Dysfunctional or lacking CFTR protein in cholangiocytes leads to a disrupted Cl^−^ and HCO_3_^−^ secretion, and a hyperviscous bile. The resulting ductular biliary obstruction and portal inflammation may first cause a focal, but later multilobular fibrosis and cirrhosis. A recent case report refers to an infant with CF presenting with neonatal cholestasis mimicking biliary atresia, in whom a new *CFTR* mutation (c.3871 G>T) resulting in a severe phenotype has been described [53]. Among a range of candidate genes, SERPINA-1Z–allele has been identified as a modifier gene for the development of CFLD [45].

### 5.2. Impact of Lipid and Glucose Homeostasis

Low BMI, abnormal nutritional parameters, growth failure associated with exocrine pancreatic insufficiency (pancreatic atrophy with lack or reduction of digestive enzyme secretion leading to fat malabsorption), and CF-related diabetes (CFRD) have been linked to the development of CFLD, all conditions that might be related to dyslipidemia (normal to low total cholesterol, low high density lipoprotein (HDL), low density lipoprotein (LDL), high triglycerides). In fact, hypercholesterolemia and hypertriglyceridemia are found in about 8% to 15% of non-transplanted CF patients [54,55]. Total cholesterol (but also HDL), and triglycerides rise with increasing age and BMI [56]. However, Prevalence of hypercholesterolemia (15% *vs.* 32%) and hypertriglyceridemia (8% *vs.* 42%) significantly increases after lung transplantation implicating a role for immunosuppressive medication [55]. Nevertheless, very little is known about alterations in lipid homeostasis in CF prior to and after lung transplantation.

### 5.3. The Role of Bile Acids

Although data are partly conflicting, BAs have been linked to the pathogenesis of CFLD already thirty years ago. Bile acids (BA) are inherently cytotoxic, and retention of BA in CF may lead to progressive liver fibrosis. The concentration of BA in serum of CFLD patients was shown to be significantly elevated compared to patients without CFLD, as well as healthy controls [57]. Additionally, BA composition is altered in CF patients, showing higher levels of the primary BA cholic acid and chenodeoxycholic acid. Serum cholic acid levels were associated with hepatic fibrosis, inflammation and limiting plate disruption [58]. In a study in CF patients without CFLD, total fecal BA levels were markedly increased compared to healthy controls showing a selective malabsorption of cholic acid [59]. O’Brien *et al.* reported a higher fecal BA loss in CF patients without liver disease as compared to patients with CFLD, and suggested a defect in the ileal absorption of BA within the enterohepatic BA circulation [60]. However, studies investigating the mechanisms at the basis of BA malabsorption and altered enterohepatic BA circulation in CF patients are lacking.

Farnesoid X receptor (FXR), expressed in liver and intestine, represents a major regulator of BA homeostasis. As the main BA receptor, FXR regulates BA synthesis by a negative feedback loop. Upon activation by BA, FXR binds as a heterodimer with retinoid X receptor (RXR) to the FXR responsive elements regulating the transcription of genes involved in bile salt synthesis, transport to the liver, and metabolism in the liver and intestine (in the liver via cholesterol 7-α-mono-oxygenase or cytochrome P450 7A1 (CYP7A1), bile salt export pump (BSEP), organic solute transporter heterodimer (OSTα/β), and short heterodimer partner (SHP); in the intestine via apical sodium-dependent bile acid transporter (ASBT), ileal bile acid binding protein (IBABP), SHP, and OSTα/β). BA activated FXR induces the expression of FGF19 (FGF15 = orthologue in mice) mainly in enterocytes, but also in human hepatocytes. Intestinal FGF19 functions as an enterokine that signals the presence of BA in the intestine to the liver. This signal transduced by fibroblast growth factor receptor (FGFR) 4 and the involved protein cascade suppresses CYP7A1, the rate limiting enzyme of BA synthesis in the liver [61,62].

Impaired FXR signaling is associated with increased hepatocellular damage upon bile acid challenge [63]. Of note, FGF19 was shown to be ectopically expressed in livers of cholestatic patients, representing an adaptive response reducing disease progression [64]. Selective activation of FXR in the intestine protects mice against cholestasis by activation of FGF15 (mouse ortholog of FGF19) via a negative feedback loop to hepatic bile acid synthesis [65]. FXR exerts its anti-inflammatory effects among others via inhibition of hepatocellular nuclear factor κB (NF-κB) activation and induction of tumor necrosis factor α (TNFα) inducible nitric oxide synthase and cyclooxygenase-2 [66]. Under the condition of biliary obstruction mice lacking FXR have induced intestinal inflammation and bacterial overgrowth [65].

Recently, Ho *et al.* identified genetic variants in the sodium-dependent apically located bile acid transporter system (ASBT) in healthy individuals, the transporter to which BA bind to be reabsorbed in the terminal ileum, consisting of functional SNPs altering taurocholate transport *in vitro* [67]. Furthermore, reports on patients with progressive familial intrahepatic cholestasis (PFIC), a disease characterized by cholestasis starting in infancy and cystic fibrosis-like extrahepatic manifestations within the pancreas, have shown mutations in different genes being involved in the regulation of BA homeostasis. In *PFIC2* mutations within *ABCB11*, the gene encoding for the canalicular bile salt export pump (BSEP), lead to a defect in the extrusion of bile salts across the canalicular membrane of hepatocytes causing cholestasis [68]. In PFIC1, a gene defect in ATP8B1 encoding the protein FIC1 which is responsible for mediating amino phospholipid translocation in plasma membranes was shown to cause deregulations in bile salt transporters through decreased expression and/or activity of FXR. A defect of ATP8B1 along with CFTR downregulation led to impaired bile secretion of cholangiocytes in humans [69].

Recently, investigating *CFTR^−/−^* and *F508del* mice, Debray *et al.* described alterations in gall bladder emptying. This may be mediated by an overexpression of the myorelaxant vasoactive intestinal peptide (VIP) in gallbladders [70]. Furthermore, the amount of secondary BA was lower, and fibroblast growth factor-15 (FGF15) and BA transporters were lower in the ileum but higher in the gallbladders of *CFTR^−/−^* mice, compared with wild-type mice, whereas enzymes that synthesize BA were down-regulated in livers of *CFTR^−/−^* mice. Cholecystectomy partially reversed changes in gene expression and partially restored levels of secondary BA. The authors therefore hypothesized the existence of a so-called cholecystohepatic shunting, based on a disrupted enterohepatic circulation of BA due defects in gallbladder emptying, thereby restricting the amount of toxic secondary BAs that enter the liver. If this is also true in humans remains to be elucidated.

Furthermore, growing evidence suggests a protective effect of biliary HCO_3_^−^ secretion against BA induced bile duct damage, the so-called HCO_3_^−^ umbrella [71]. Mutations in *CFTR* causing a dysfunctional or lacking CFTR protein causes altered HCO_3_^−^ secretion and may contribute to the formation of aggressive bile juice harming cholangiocellular epithelium. *In vitro* studies aside the setting of CF using human cholangiocytes have indicated that keeping an alkaline pH by HCO_3_^−^ secretion might prevent protonation of apoptosis, inducing BA, such as glycochenodeoxycholate (GCDC) [4,72,73]. The Cl^−^/HCO_3_^−^ exchanger AE2 (SLC4A2) is regarded the major mediator of HCO_3_^−^ secretion in human cholangiocytes [73]. However, suitable *in vivo* mouse models to evaluate the model of an HCO_3_^−^ umbrella in humans, however, are lacking [74].

Due to their recently-discovered hormonal functions, BA also act as metabolically active molecules regulating not only lipid digestion but also hepatic lipid metabolism. As such, BA are important regulators of hepatic de novo lipogenesis [75], which was recently reported to be impaired in an animal model of CF [76]. Therefore, impaired BA homeostasis could also contribute to hepatic steatosis and lipotoxicity in the progression of CFLD. Alterations in plasma and tissue fatty acid profile, in particular decreased polyunsaturated fatty acids (PUFA; *i.e.*, linoleic acid, arachidonic acid, *etc.*), are widely recognized in CF [77]. Similar fatty acid profiles have been linked to the pathogenesis of NAFLD [78]. Of note, CF associated fat malabsorption seems not to be related to CFTR depletion [79]. Thus far, the causes of frequently observed steatosis in CF patients are unsettled. Of note, FXR and its downstream targets (SHP, liver receptor homologue-1 (LRH-1), liver X receptor (LXR), sterol regulatory binding protein-1c (SREBP-1c)) are also involved in the regulation of hepatic lipogenesis and triglyceride metabolism [80]. *FXR^−/−^* mice lacking this regulatory mechanism present with a phenotype of steatosis and hypertriglyceridemia [81]. In turn, ligands of FXR have shown promising results in animal models of NAFLD and clinical pilot studies [80]. Furthermore, in mice treated with BA, fructose-induced hepatic steatosis could be markedly attenuated [82]. This protective effect was, among other things, associated with downregulation of SREBP-1 and fatty acid synthase (FAS) mRNA expression.

However, function of the mentioned BA transporters and receptors and their relation to CFLD have not yet been studied in CF patients.

### 5.4. The Role of Intestinal Inflammation and Changes in the Gut Microbiom

Recently, an association between the development of NAFLD and intestinal inflammation, gut permeability and intestinal microbiota was demonstrated [83,84]. The relation of alterations in the gut on the one hand, and CFLD on the other hand, have not been investigated thus far. However, apart from liver dysfunction, high numbers of CF patients present with intestinal disorders. Approximately 85% of patients suffer from PI with impaired digestion presenting as steatorrhea, fat-soluble vitamin deficiency, and poor nutritional status. Importantly, regardless of adequate pancreatic enzyme replacement therapy, a majority of patients with CF suffer from abdominal pain and altered gastrointestinal motility suggesting CF related gastrointestinal disease [85,86,87]. Other intestinal diseases, such as Crohn’s disease and celiac disease have been shown to occur with increased frequency in CF [88,89].

One of the major causes attributed to CF associated intestinal disorders is small intestinal bacterial overgrowth (SIBO) [90], which can be found in one to two thirds of CF patients [91]. Evidence to support the presence of alterations in gut microbiota has been reported also in murine models of CF that have a pronounced increase in bacterial load in the small intestine [92,93]. To date, even if the preclinical data support the fact that SIBO induces intestinal inflammation and malnutrition that can be treated by antibiotics, these findings have not been reproduced in patients, nor has screening for SIBO been included in the routine management of patients with CF [94]. Antibiotic treatment reduced the expression of pro-inflammatory genes in the intestine of CF mice, supporting the hypothesis that SIBO represents a significant factor for intestinal inflammation in CF mice [92]. As an important modifiable factor, also treatment with probiotics and prebiotics have shown promise in improving CF gastrointestinal disease [95]. A recent prospective double blind cross over study in CF patients could demonstrate a positive effect of Lactobacillus reuteri on the gut microbiota, thereby significantly decreasing gastrointestinal inflammation and reducing digestive discomfort [96].

The BA receptors FXR and the G-protein coupled bile acid receptor 1 (TGR5, GPBAR1) are important regulators, not only of BA homeostasis, but also gut barrier function. Impaired FXR and TGR5 signalling is not only associated with hepatic cholestasis, but also intestinal inflammation and bacterial overgrowth. Feeding taurodeoxycholic acid induced mucosal enterocyte proliferation and decreased apoptosis in wild type, but not in FXR knockout mice. Thus, luminal bile salts exhibit positive effects in keeping the gut barrier and preventing bacterial translocation [97]. Recently, Torres *et al.* showed that FXR expression is decreased in colonic mucosa of patients with PSC and colitis-associated neoplasia [98]. FXR also regulates intestinal microbial defence and has a crucial role in limiting bacterial overgrowth. The FXR agonist GW4064 was shown to repress bacterial overgrowth and bacterial translocation, and to attenuate mucosal injury [65]. As mentioned before, selective activation of FXR in the intestine protects mice against cholestasis by activation of FGF15 (mouse orthologue of FGF19) via a negative feedback loop to hepatic bile acid synthesis [65]. TGR5 is expressed in many different organs like several liver cells, intestine, monocytes, brown adipose tissue, skeletal muscle and areas of the central nervous system. Stimulation of TGR5 improves not only glucose homeostasis, reduces steatosis, but also has potent anti-inflammatory effects reducing the expression of pro-inflammatory cytokines upon stimulation [99,100]. Stimulation of isolated rat Kupffer cells by TGR5 agonists resulted in reduced production of IL-1, IL-6 and TNFα in response to LPS exposure [101]. In addition, TGR5 could be identified to regulate intestinal integrity in an animal model of inflammatory bowel disease [102]. The dual FXR/TGR5 agonist INT-767 was shown to reduce hepatobiliary injury in an *Mdr2^−/−^* mouse model of chronic cholangiopathy by reduction of biliary BA output and promotion of biliary HCO3^−^ secretion [103].

### 5.5. The Impact of Maintenance Immunosuppression after Lung Transplantation

Since life expectancy of CF patients is increasing due to improved patient care and lung transplantation, optimizing patient care also after lung transplantation gains importance. There is increasing evidence that liver abnormalities, such as steatosis (in combination with diabetes, arterial hypertension and hyperlipidemia) are part of a metabolic syndrome induced by immunosuppressive regimens including CNI and steroids [104,105]. How this refers to the development of steatosis or steatohepatitis in patients with CF, frequently suffering from CF related diabetes and hyperlipidemia already prior to transplantation, and possible differences in terms of NAFLD have not been investigated thus far.

Little is known about the effects of maintenance immunosuppression including calcineurin inhibitors (CNI) on BA homeostasis in humans. However, cyclosporine A (CSA) has been shown to interact with various steps of bile salt metabolism *in vitro* and *in vivo*. It was shown to inhibit bile salt synthesis in cultured rat and human hepatocytes and to lead to reduced bile flow in an animal model [106,107]. CSA competitively inhibited sodium dependent uptake and hepatobiliary secretion of radiolabelled taurocholate in animal models [108,109]. In addition, impaired ileal BA absorption was reported after ileal perfusion with CSA in rats [110]. Finally, Hulzebos *et al.* reported effects of chronic CSA treatment on enterohepatic BA circulation. It markedly reduced cholate synthesis in rats without changes to the cholate pool size suggesting that CSA enhances efficacy of intestinal cholate reabsorption through increased ASBT protein expression in the distal ileum [111].

How CNI including tacrolimus, frequently used after lung transplantation, influence enterohepatic BA circulation in CF and non CF patients is unknown. However, in liver transplant recipients due to other diseases than CFLD, three months after transplantation at least no differences in BA composition in patients treated with tacrolimus compared to CSA were found [112].

## 6. Treatment Options for CFLD

### 6.1. Medical Treatment Options

Currently, the naturally occurring hydrophilic bile acid (BA) ursodeoxycholic acid (UDCA) is the only drug available for the treatment of CFLD [113]. In contrast to PBC, where UDCA is the established treatment of choice [114,115] CF patients treated with UDCA show improvement of liver function tests and liver morphology, yet without impact on survival [8]. A recent systematic Cochrane review highlighted the paucity of randomized controlled trials (RCTs) of UDCA use in CFLD and concluded that there is insufficient evidence to justify its routine use in CFLD. Only three RCTs of the use of UDCA for at least three months in 118 participants could be included in this review. Adverse effects were rare with UDCA, but long-term relevant outcomes such as the need for liver transplant, death or effects on portal hypertension were not adequately addressed in any study [116]. In a dose-response study, Colombo *et al.* demonstrated that the biochemical response to UDCA was best with a dose of 20 mg/kg/day [117]. However, recent data gave rise to concerns about the safety of UDCA, as use of high dose UDCA (28–30 mg/kg/day) was associated with an increased risk of malignancy and significantly more adverse events in PSC [118,119]. Targeting of BA signaling pathways could be an attractive option for treatment of intestinal and hepatic disease. BA derivatives (FXR ligand INT-747, dual TGR5/FXR agonist INT-767, nor-UDCA) have shown promising results in the treatment of cholestatic liver diseases [103,120].

Recently, ivacaftor, an orally bioavailable potentiator of CFTR with particular efficacy in gating (class III) mutations (where the protein is expressed but does not open and close normally) was approved for patients with *G551D* mutation, and showed promising results in improving lung function [121,122,123]. Data on its effect on CFLD are missing. Effects of personalized mutation specific therapies that potentiate or correct CFTR function on the hepatobiliary tract are eagerly awaited [124].

### 6.2. Liver Transplantation

In general, treatment of cirrhosis complications in CFLD does not differ from other liver diseases. With regard to portal hypertension, use of β-blockers is limited due to concomitant CF lung disease. Liver transplantation or combined liver-lung-transplantation [125,126,127] is a therapeutic option for advanced decompensated cirrhosis (falling albumin < 30 g/L, increasing coagulopathy not correctable by vitamin K, ascites, jaundice, intractable variceal bleeding, or encephalopathy) but also hepatopulmonary and portopulmonary syndrome, deteriorating pulmonary function (FEV1/FVC < 50) and severe malnutrition unresponsive to intensive nutritional support [8]. Although impaired pulmonary function is thought to negatively influence the outcome of liver transplantation in CF, several reports have demonstrated improvement of respiratory function and general condition after isolated liver transplantation [128]. Recently similar outcomes for isolated liver *vs.* combined liver-lung transplantation in CF were reported [127]. Contraindications to isolated liver transplantation in favor of combined liver-lung transplantion in CF include longstanding history of severely compromised lung function with frequent exacerbations of pulmonary infections, colonization with Burkholderia cepacia or other multidrug-resistant organisms, raised resting arterial pCO_2_, extensive pulmonary fibrosis on chest computerized tomography, and severe pulmonary hypertension with right ventricular dysfunction [126]. Of note, long-term outcomes in patients with CFLD are reported to be inferior in comparison to those undergoing liver transplantation for other etiologies, which might be due to impaired nutritional condition, worsening pulmonary status and infection in CF [129,130].

## 7. Conclusions

CFLD is an under-recognized and under-treated organ manifestation of CF. Diagnostic screening tools, such as imaging studies and minimally-invasive biomarkers for the detection and surveillance of CFLD have to be refined. Since the treatment options are limited due to a lack in understanding the underlying pathomechanisms further studies on the effects of BA homeostasis and circulation and the influence of the gut microbiome on CFLD development should be encouraged.
